# SIDMA as a criterion for psychiatric compulsion: An analysis of compulsory treatment orders in Scotland

**DOI:** 10.1016/j.ijlp.2021.101736

**Published:** 2021

**Authors:** Wayne Martin, Miriam Brown, Thomas Hartvigsson, Donny Lyons, Callum MacLeod, Graham Morgan, Lisa Schölin, Kathleen Taylor, Arun Chopra

**Affiliations:** a Essex Autonomy Project, School of Philosophy and Art History, University of Essex, Wivenhoe Park, Colchester, Essex CO4 3SQ, UK; b Mental Welfare Commission for Scotland, 91 Haymarket Terrace, EH12 5HE Edinburgh, UK

**Keywords:** Psychiatric compulsion, Impaired decision making, Mental health legislation, Capacity, Scotland, Insight

## Abstract

Scottish mental health legislation includes a unique criterion for the use of compulsion in the delivery of mental health care and treatment. Under the Mental Health (Care and Treatment) (Scotland) Act, 2003, patients must exhibit ‘significantly impaired decision-making ability’ (SIDMA) in order to be eligible for psychiatric detention or involuntary psychiatric treatment outside the forensic context. The SIDMA requirement represents a distinctive strategy in ongoing international efforts to rethink the conditions under which psychiatric compulsion is permissible. We reconstruct the history of the Scottish SIDMA requirement, analyse its differences from so-called ‘fusion law,’ and then examine how the SIDMA standard actually functions in practice. We analyse 100 reports that accompany applications for Compulsory Treatment Orders (CTOs). Based on this analysis, we provide a profile of the patient population that is found to exhibit SIDMA, identify the grounds upon which SIDMA is attributed to individual patients, and offer an assessment of the quality of the documentation of SIDMA. We demonstrate that there are systemic areas of poor practice in the reporting of SIDMA, with only 12% of CTOs satisfying the minimum standard of formal completeness endorsed by the Mental Welfare Commission. We consider what lessons might be drawn both for the ongoing review of mental health legislation in Scotland, and for law reform initiatives in other jurisdictions.

## 1 Introduction

We are living through a period of intensive reassessment of the legal standards and mechanisms used to authorise involuntary medical treatment, particularly in the context of mental illness or impaired capacity. Within the UK, this reconsideration has led to an innovative new statute in Northern Ireland, an independent review of the *Mental Health Act* in England and Wales, and the commissioning of a root-and-branch review (ongoing at the time of writing) of mental health and mental capacity law in Scotland (Mental Capacity Act (Northern Ireland), 2016; Department of Health and Social Care, 2018; Dhanda, 2007; Secretariat to the Review of the Mental Health Legislation in Scotland, 2019). This paper contributes to these continuing discussions and debates by scrutinising a distinctive provision of Scots law in this arena: the so-called SIDMA requirement in current Scottish mental health legislation. ‘SIDMA’ is an acronym; it stands for *significantly impaired decision-making ability*, specifically in connection with ability to make decisions about medical treatment. It is a concept unique to Scots law.^2^ In the Mental Health (Care and Treatment) (Scotland) Act, 2003 (hereafter: the Act), SIDMA plays a role in several provisions regarding the exercise of coercive power in the context of mental disorder, including (but not limited to): the criteria for emergency detention in hospital (*sec*. 36, up to 72 h detention); the criteria for short term detention in hospital (sec. 44, up to 28 days detention); and provisions regarding applications for compulsory treatment orders (CTOs) (secs. 57 and 64). For both emergency and short-term detentions under the Act, the assessor must believe that the person is likely to have SIDMA; for CTOs the person must be shown to have SIDMA.

In what follows, we describe and critically assess the current practice of SIDMA-assessment in Scotland. We begin by reviewing the history of SIDMA, which finds its origins in a 2001 report that informed the drafting of the Act. Returning to that report and its reception, we identify both the policy objectives that SIDMA was designed to achieve and the rationale for adopting SIDMA as the means for achieving those ends. We then review a 2010 study which examined the operation of SIDMA in practice, and report on our own (partial) replication of this study ten years on. Our replication study takes the form of an analysis of medical reports for CTO applications held by the Mental Welfare Commission for Scotland (hereafter: the Commission). Our main aims in that analysis are threefold: (a) to examine the grounds on which persons are currently deemed to have SIDMA; (b) to compare recording practices regarding SIDMA in 2019 with the practices reported in 2010; and (c) to provide a critical evaluation of the quality of current SIDMA-reporting by assessing the extent to which current practice conforms to guidance. We also used this as an opportunity to expand upon the 2010 study in line with recent developments in human rights by assessing the extent to which practices of supported decision-making are reflected in these formal assessments of decision-making ability.

Domestically, we hope that our findings will help improve current practice under the Act and inform ongoing deliberations about whether and how it should be amended. Internationally, our aim is to raise awareness of both the promise and the pitfalls associated with an understudied approach to mental health law reform, and to assess a distinctive strategy intended to deliver mental health care and treatment (including coercive interventions when necessary) while increasing respect for patient autonomy.

## 2 Background

Shortly before the opening of the newly devolved Scottish Parliament in 1999, the Scottish Government commissioned a review of the *Mental Health (Scotland) Act 1984*. The outcome of the review is known colloquially as ‘The Millan Report’; formally its title was: *New Directions: Report on the Review of the Mental Health (Scotland) Act 1984* (Scottish Executive, 2001a; hereafter: Millan).^3^ One of the ‘new directions’ recommended in the report was the addition of a new criterion for authorising compulsion in care and treatment for mental disorder. The Millan Report did not itself use the term ‘SIDMA,’ but it proposed that compulsion be used in the delivery of mental health care outside forensic contexts only if:

as a consequence of the person's mental disorder, the person's judgement is impaired to a nature or degree which would justify compulsory measures. (Millan, 63).

We shall refer to this as *the impaired judgement condition*. The Millan Committee proposed that this new criterion should function as an additional prerequisite for compulsion – over and above the requirements that are familiar from earlier legislation and other jurisdictions: the presence of mental disorder of a nature or degree that requires treatment, risk of harm to the patient or to other persons.

The Millan Committee's innovative recommendation was motivated in part by two widely shared policy objectives: promotion of patient autonomy and avoidance of discrimination. Both autonomy and non-discrimination figure prominently in the ‘recommended principles’ that the Millan Committee enumerated at the head of its report. As regards autonomy, the Committee stated that ‘[w]herever possible care, treatment and support should be provided to people with mental disorder without recourse to compulsion’ (Millan, 19). As regards non-discrimination, the Committee held that ‘[p]eople with mental disorder should whenever possible retain the same rights and entitlements as those with other health needs’ (Millan, 18).

In looking for ways to advance these policy objectives, one widely discussed strategy has come to be known as *fusion law* – so-called because it involves ‘fusing’ elements of mental capacity law and mental health legislation. To understand what is distinctive about fusion law, we must keep in mind that Scotland's *Mental Health Act 1984*, like most mental health statutes around the world, was indifferent to a patient's decision-making ability. The criteria for the use of psychiatric coercion focused on illness and risk; the question of whether the patient had the ability to make his or her own decision about treatment was not part of the statutory formulation.^4^ Advocates for fusion law, by contrast, propose to reserve psychiatric compulsion for patients who lack the mental capacity to make their own treatment decisions. Such an approach, it has been argued, serves both to increase patient autonomy (by allowing patients to make their own treatment decision whenever they are able to) and to decrease discrimination (by bringing the legal framework for mental health treatment into closer alignment with the legal basis for the treatment of physical illness or injury).^5^

The Millan Committee's endorsement of the impaired judgement condition is best understood as a kind of compromise with fusion law. In their review, the Committee *considered but rejected* the inclusion of a capacity test among the conditions for use of compulsion. The Committee's view was that while such an approach might have a number of advantages, there were also ‘strongly held views against making a capacity test the fundamental criterion for intervention’ (Millan, 55). They reported on concerns that such a framework ‘might discourage early intervention’ (Millan, 56), and indicated that ‘[m]any of our respondents also felt it to be wrong that a person with a mental disorder would be allowed to bring severe harm to himself or herself, perhaps including death, on the basis of a judgement that the person had the capacity to make such a decision’ (Millan, 56). The impaired judgement standard was therefore proposed as a kind of compromise – adding a new prerequisite for the use of compulsion but stopping short of ‘full fusion’.

The Millan Report did much to shape debate in the new Scottish Parliament. Scottish legislators followed Millan's recommendation in plotting a middle path between the status quo ante and a full fusion approach. But in doing so, they also took a further step closer to the fusion law approach. Where the Millan recommendation spoke of *impaired judgement*, the Scottish Government explicitly introduced the concept of *impaired decision-making ability* into the criteria for compulsion.^6^ The final statutory language does not adopt the kind of capacity test backed by fusion law proponents; instead, the new condition was SIDMA. As regards eligibility for emergency detention, for example, the law requires that:

because of the mental disorder, the patient's ability to make decisions about the provision of medical treatment is significantly impaired. (*sec*. 36(4)).

The same language was included in the statutory conditions for short-term detention in hospital (Mental Health (sec. 44) and for the issuance of a CTO (secs. 57 and 64).

Two details of the legal provisions that established SIDMA will be particularly important in what follows. Notice first the use of the words ‘because of’ in the statutory language pertaining to SIDMA. The new criteria for compulsion introduced with the Act require not only the *presence* of SIDMA; they also require that SIDMA be *caused or explained* by the person's mental disorder or impairment. But what would be particularly significant for the subsequent implementation of SIDMA is a second feature of the final legislation: the Act *offers no definition of SIDMA*. The consequence: the new statute left considerable scope for interpretation in applying the new prerequisite.

It is important to keep in mind that there is a statutory definition of decision-making incapacity in Scots law. The Adults with Incapacity (Scotland) Act, 2000 (hereafter: AWIA), adopted just two years before the Act, includes such a definition.^7^ But both the *Training Manual* and the *Code of Practice* for the new Act make it clear that “SIDMA is not the same as ‘incapacity’ under the Adults with Incapacity (Scotland) Act, 2000” (Scottish Executive, 2005a, 45; see also Scottish Executive, 2005b, para. 1.22–24). This in turn yields a further challenge for practitioners, who are expected to apply two legal standards pertaining to decision-making ability: the standard defined in the AWIA and the undefined concept of SIDMA in the Act.

In the years since the adoption of the Act, a variety of attempts have been made to provide practitioners with the guidance they need in order to apply the SIDMA standard consistently in practice. The *Training Manual* associated with the new legislation explained the concept of SIDMA as follows:

SIDMA occurs when a mental disorder affects the person's ability to believe, understand and retain information, and to make and communicate decisions. It is consequently a manifestation of a disorder of mind. SIDMA arises out of mental disorder alone; ‘incapacity’ can also arise from disease of the brain or impaired cognition, and can include physical disability. SIDMA is not the same as limited or poor communication, or disagreements with professional opinion. (Scottish Executive, 2005a, p.45; see also Scottish Executive, 2005b, para. 1.22).^8^

The *Code of Practice* indicated that SIDMA refers specifically to ‘a significant impairment with respect to decisions about the provision of medical treatment for mental disorder’ (Scottish Executive, 2005b, para. 1.25). It also echoed the language of the *Manual* in emphasising that SIDMA ‘is not the same as having a problem communicating or disagreeing with professional opinion’ (Scottish Executive, 2005b, para. 1.27).

Five years after the 2003 Act came into force, Shek, Lyons, and Taylor (2010) undertook an analysis of CTOs that had been initiated under the new legislation. Their study analysed the SIDMA field on 100 anonymised CTOs held by the Commission pursuant to the Commission's statutory role of monitoring and promoting best practice in the use of the Act. The study aims were partly descriptive and partly normative. Descriptively, their study sought to identify the most common grounds that were cited by assessors in support of a finding of SIDMA. Normatively, they assessed the adequacy of the cited evidence, identifying both best and worst practice. The 2010 study thus gave an important first portrait of how this innovative but undefined Scottish legal concept was actually being applied in psychiatric practice.

One finding of the 2010 study was that impaired insight featured frequently among the reasons cited by assessors in their finding of incapacity (Shek et al., 2010, 239). ‘Insight’ is a concept that occurs frequently in psychiatric discourse, but which has also been the subject of controversy.^9^ Although it lacks an agreed formal definition in medicine or law, it is commonly used to refer to a patient's self-awareness of illness or impairment. A patient who is aware that they are living with a mental disorder and who is able to identify at least some aspects of their condition as symptoms will commonly be described in clinical discourse as ‘having insight.’ A patient who is ill but lacks awareness of their illness will commonly be described as ‘lacking insight’ or as having ‘impaired insight’ (Oyebode, 2018; Sims, 2002). Shek and colleagues found that impaired insight was the single most commonly cited ground for a finding of SIDMA, appearing in 58% of the analysed CTOs; in 44% of cases, impaired insight was the *only* reason cited as evidence of SIDMA. Other grounds cited in support of a finding of SIDMA included limited cognitive function (13%) and the presence of psychotic symptoms (24%).^10^

On the basis of their study, Shek et al. made a number of recommendations pertaining to SIDMA. They recommended that CTOs should indicate ‘the actual reasons for SIDMA,’ and that assessors should include in their reports an explanation of ‘how the individual's mental disorder … affect[s] their ability to make decisions about treatment’; assessors should not simply record the condition or disorder. Where impaired insight is thought to be a factor in SIDMA, Shek and colleagues called for assessors to explain how lack of insight affects the person's decision-making ability.^11^ Finally, they proposed that an adequate justification of a finding of SIDMA should specify whether ‘the individual [is] able to understand, retain, make and communicate decisions about treatment, and if not, why not’ (Shek et al., 2010, 242).

The fifteen years since the Act came into force has been an exceptionally active period in the law and practice surrounding the assessment of decision-making ability. Just as the Act came into effect in Scotland in 2005, the Westminster Parliament adopted the Mental Capacity Act 2005 (hereafter: the MCA) for England and Wales, providing its own statutory definition of mental incapacity, and setting up a specialist Court of Protection. Internationally, the UN adopted *Convention on the Rights of Persons with Disabilities* (UNCRPD) in 2006, which was ratified by the UK in 2009. Both the MCA and the UNCRPD contained provisions that called for steps to be taken to support people to make their own decisions wherever possible. (Mental Capacity Act 2005, *sec*. 1(3); UN General Assembly, 2006, art. 12(3)) Some have called for a ‘paradigm shift’ from so-called ‘substitute decision making’ to ‘supported decision-making.’ (Bach & Kerzner, 2010; Bartlett, 2012; Dhanda, 2007; UN Committee on the Rights of Persons with Disabilities, 2014). There has therefore been intensive interest in techniques for supporting persons with impaired decision-making ability to make their own decisions, and to recover decision-making abilities when these are lost or impaired (Mental Welfare Commission for Scotland, 2017a).

## 3 Methods

### 3.1 Sample

Following the approach taken by Shek et al. (2010), we extracted 100 con*sec*utive CTOs from the records held by the Commission. The Commission is a non-departmental governmental body which has a statutory duty to monitor the use of the Act and to promote best practice (sec 5). As part of that role, all detentions under the Act must be reported to the Commission. Our analysis covered CTOs with a starting date between January and February 2019.

Applications for a CTO are made by a Mental Health Officer (MHO) – a social worker with additional training in mental health and illness – and are accompanied by two reports made by two medical practitioners (sec 57). Ideally one should be provided by an Approved Medical Practitioner (AMP) and the other by a General Practitioner (GP). An AMP is either a member or fellow of the Royal College of Psychiatrists or a medical practitioner with four years whole time equivalent working in mental health services. This is in keeping with the expectation that the GP might provide longitudinal knowledge of the patient and the psychiatrist particular expertise around the current episode of illness (Scottish Executive, 2005b, para. 3.11). The forms are identical and require the practitioner to state how well they know the patient. From the list of forms submitted to the Commission, we alternated between the first and second form listed in support of an application. Where a GP is unavailable the second report may be provided by another AMP, subject to ensuring that there are no conflicts of interest.

From routine information completed on the form, we extracted basic demographic characteristics (age, gender, and diagnosis) of the person being assessed, whether the clinician completing the form was a GP or an AMP, the clinician's familiarity with the patient, and risk of harm to the patient and/or others. We categorised this information in the same manner as in the study by Shek et al. (2010) in order to facilitate relevant comparisons. As part of the application for a CTO, the clinician also has to provide a reason for the detention and justify why the patient is considered to have SIDMA. We transcribed all information from the part of the form containing the SIDMA justification.

### 3.2 Data analysis

Following transcription of all 100 forms, a coding framework was developed by the project team, led by MB, to provide a structure for coding information provided in support of the claim that the patient exhibited SIDMA. The coding framework was initially drafted using categories from Shek et al., 2010 and Mental Welfare Commission for Scotland, 2017b. To reflect progression in human rights-based approaches to mental health law through the ratification of the UNCRPD in 2009, we also recorded whether the forms noted any evidence of supported decision making. Although the UNCRPD was ratified after the Act came into practice, the Act is underpinned by principles which include participation wherever possible (*sec*. 1(3)(c)). The coding framework was piloted by MB on ten forms and discussed with AC and the wider project team; this exercise was repeated at 20 forms.

Conducting the pilot coding exercises revealed that there were findings of SIDMA that were not captured in the categories identified by Shek et al., 2010. Some assessors cited the inconsistency of the patient's decision-making as a basis for a finding of SIDMA; others included treatment non-adherence or treatment non-concordance as a basis. We also found examples of what can be described as ‘proxy’ reasons such as the presence of a guardianship arrangement or a power of attorney that were taken to imply that the person being assessed had SIDMA. For the full coding exercise, we adopted the expanded coding framework as presented in the Supplementary File.

The expanded coding framework was then applied to the data set. Lack of insight had emerged as the biggest single factor in the previous study. Forms were coded as citing insight as a ground for a finding of SIDMA if one or more of four conditions were met: (a) the form explicitly used a form of the word ‘insight’ (‘insight,’ ‘insightless,’ ‘insightlessness,’ etc); (b) the SIDMA field of the form described the patient as not believing, accepting or agreeing that they have a mental health problem; (c) the SIDMA field of the form described the patient as failing to comprehend or understand their mental health problem; (d) the SIDMA field described the patient as failing to recognise the value of treatment (Gurbai, Fitton, & Martin, 2020). All cases that were coded for ‘insight’ as a ground for a finding of SIDMA were independently coded by a second coder (TH); discrepancies were resolved through discussion between MB, AC, TH and WM. All analysis was conducted in Excel and descriptive frequency statistics were calculated for each of the relevant codes.

### 3.3 Quality assessment

Like Shek et al., our aims in conducting the analysis of CTOs were both descriptive and normative. In assessing the normative adequacy of the SIDMA fields, we adopted our standard from a 2017 Commission paper that provides guidance on the assessment of SIDMA in the context of eating disorders (Mental Welfare Commission for Scotland, 2017b). We deemed the SIDMA field to be formally complete only if it met all three of the following conditions: (a) it reported on specific symptoms of a mental disorder; (b) it indicated which component(s) of the overall decision-making process was impaired (e.g., the abilities to understand, retain, use and weigh); and (c) it indicated that there was a link between these first two elements, as required under the ‘because of’ clause in the statutory requirement. We also calculated the readability of each of the SIDMA fields, using the Flesch Reading Ease (Farr, Jenkins, & Paterson, 1951; hereafter: FRE) score as a proxy indicator of the likelihood that the text in the SIDMA field would be readily intelligible to patients and others who support them. The use of accessible language in CTOs is a key consideration in terms of the ‘participation’ of the individual, which is one of the principles of the Act.

## 4 Results

**Fig. 1:**
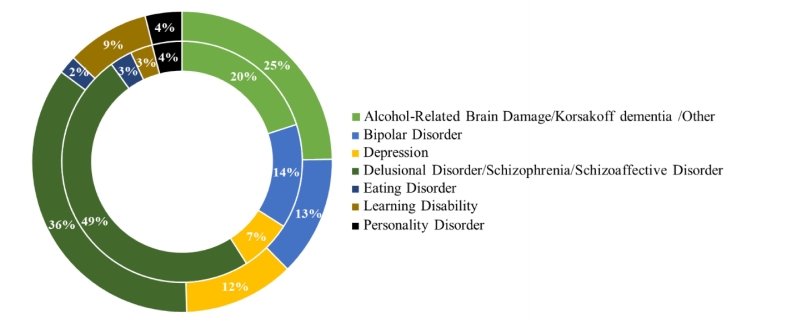
Diagnosis of patients (Shek et al. inner circle; current study outer circle).
c) Reasons Reported for SIDMA: In their 2010 study, Shek and colleagues reported four categories of reasons for SIDMA: lack of insight, confusion/cognitive impairment, psychotic symptoms and other reasons (including severe depression and learning disability). In our sample we found a similar percentage of forms where the clinician noted psychotic symptoms as reason for SIDMA to the previous study (21% and 24%, respectively). As regards the inclusion of lack of insight as the basis for a finding of SIDMA, we found an even higher rate than was reported in the 2010 study: 76% cited lack of insight, compared to 58% reported by Shek and colleagues in 2010 (Table 1). In our study, 62 forms provided more than one reason for SIDMA, compared to only 16 in the previous study. In our analysis we identified five forms in which no evidence of SIDMA was cited; no cases of this kind were reported by Shek and colleagues.

a) The Assessors: The majority of the 100 mental health reports were completed by an AMP (87%), with a minority completed by GPs (13%). This was similar to the previous study where 86% were completed by AMPs. Of the 13 forms completed by GPs, 12 patients (92%) were known to the practitioner and one was unknown prior to the interview in relation to the CTO. In the 87 forms completed by AMPs, 34% of patients were unknown to the clinician prior to interview, 31% were known prior to the interview, 22% were known since the admission, 5% were known since transfer to the ward or the care was transferred to the clinician. Seven forms were left blank for this information.
b) Age and Diagnosis: The mean age of patients in the sample was 50 years (range 16–90 years), with 53% male patients. The main diagnoses of patients were Delusional Disorder, Schizophrenia or Schizoaffective Disorder (36%), Alcohol-Related Brain Damage, Korsakoff dementia or other (25%), depression (12%), Bipolar Disorder (13%), Learning Disability (9%), Personality Disorder (4%), Eating Disorder (2%). A comparison of the main diagnoses in the study by Shek et al. (2010) is presented in Fig. 1.

**Table 1:** ‘Reasons’ for SIDMA.

Study	Lack of insight	Confusion/cognitive impairment	Psychotic symptoms	Other^a^
Shek et al. (2010)^b^	57 (58%)	13 (13%)	24 (24%)	26 (26%)
Current study	76 (76%)	25 (25%)	21 (21%)	35 (35%)

a Other reasons include learning disability/difficulty, manic illness and mental illness, depressive symptoms, agitation, inability to communicate, anorexia/anorexic cognitions, alcohol misuse, emotional instability, consistency of thought, executive function, illicit substance misuse, Korsakoff's, obsessional interests, organic illness, and suggestibility.

b One mental health report in Shek et al. was excluded as it was unreadable (*N* = 99).
d) Quality Assessment: The 2017 guidance from the Commission indicates that SIDMA-assessors should record the symptom of the mental disorder that impacts the decision-making process (Mental Welfare Commission for Scotland, 2017b). Most of the forms (93%) noted a symptom which contributed to the individual having SIDMA, however only in 18 of these forms (18%) did the clinician identify the specific aspect of the decision-making process that was impaired. Only 12 forms indicated a clear link between the specific symptom of the disorder and the specifically affected part of the decision making process. That is, only 12% of CTOs that we analysed satisfied the minimum standard of formal completeness that we drew from the Commission's 2017 guidance.
e) Supported Decision Making: We found only four examples of supported decision making, which we interpreted in the broadest possible sense to include any steps taken to enhance the person's ability to make decisions. The main techniques recorded were repetition, rephrasing of information, or revisiting the decision at a later date. One form noted that the information had been simplified but did not expand on how that had been done.
f) Readability: The median FRE score was 45.1 (IQR = 26.4–57.7), which is significantly below the recommended target of 60 (Lim & Bennett, 2020). Only 21% of forms had a RFE of 60 or above. Interestingly, the lowest scoring forms did not tend to have a lot of medical terminology, and poor readability did not generally result from reliance on jargon. We did identify several low-readability forms that appeared to be written by the same assessor. Low scoring forms tended to have particularly long and grammatically complex sentences.

## 5 Examples

In analysing the nature and extent of the shortcomings in current practice, it is worth focusing first on the applications for CTOs in which no evidence was provided of the presence of SIDMA. The total number of cases in this category was small (just 5 in total; i.e., one in 20), but not without importance. As an example, in one of these CTO applications the SIDMA field was completed with only one sentence: ‘Mr X is unable to make decisions about his care and treatment.’ Notice that this is an *assertion* about Mr. X's inability to make decisions, but no evidence or documentation in support of that assertion is provided. Another CTO included considerably more words, but failed to provide any evidence that directly addressed the question of the person's decision-making ability. See Box 1.

Box 1 **Example of a SIDMA field that failed to provide any evidence in support of a finding of SIDMA.**

**Table:** 

*Mr X at the start of the year, despite giving a verbal agreement to remain an in-patient, left the ward contrary to that agreement and travelled to Y, necessitating police involvement in his return to the ward. He has a history, when out in the community of failing to engage with his CPN, being non-compliant with treatment and drinking to excess. This results in him becoming floridly psychotic.*

Alt-text: Box 1

The legal significance of these five cases is considerable. Under the Act, the use of compulsion in the delivery of psychiatric care outside the forensic context *requires* evidence that the patient has SIDMA. The authorisation of compulsory treatment in the absence of such evidence is therefore unlawful. The parameters of the present study did not allow us to determine whether that evidence may have been provided elsewhere – whether in other portions of the mental health reports themselves or in the context of a Tribunal hearing. But the absence of such evidence from the portion of the form devoted to SIDMA would certainly be a cause for concern.

In a second, and somewhat larger group (*n* = 12), the case for a finding of SIDMA rested *entirely* on an unelaborated and unsupported claim of impaired insight. These were particularly problematic because of the combination of three features: (i) the claim about impaired insight was the *only* rationale offered for a claim of SIDMA; (ii) no explanation was provided as to what was meant by impaired insight; and (iii) no causal or explanatory link was offered to indicate the impact of the impaired insight upon the person's decision-making abilities. See Box 2 for three examples of forms that fall into this second group.

Box 2 **Three examples of the contents of a SIDMA field in which the only evidence of SIDMA consists in an unexplained claim that the person lacks insight.**

**Table:** 

*Lacks insight into current situation and into her own mental health difficulties.*
*Mr X demonstrates little insight into the extent of his chronic mental illness, care plan and demonstrates significantly impaired decision making ability.*
*Insight fluctuates greatly - unable at times to accept advice/boundaries given for his benefit.*

Alt-text: Box 2

Responses in this category fail to conform to standards of good practice, since they neither explain what is meant by impaired insight nor attest to the ways in which the patient's impaired insight impacts upon their ability to make decisions.

A third group comprised cases in which evidence was cited in the SIDMA field, but no explanatory or causal link was established between the condition cited and the person's decision-making inability. This pattern was exhibited in just over half of the cases that we analysed. The amount of information included varied considerably within this group: in some cases the information was *very* minimal; one read in total: ‘Psychosis; no insight into illness.’ In other cases, considerably more information about the patient was provided. But in none of these cases did the record of assessment indicate the consequences of these aspects of the patient's presentation upon their ability to make decisions. See Box 3 for an example.

Box 3 **Example of the contents of a SIDMA field in which no causal or explanatory link is established to the person's decision-making abilities.**

**Table:** 

*It has been mentioned that the mental illness impacts patient concordance with treatment. There is a long history of mental illness and detained several times but still continues to relapse. The combination of issues - mental, social, drink misuse and background learning difficulty together will impact the recovery. Recently she was frequently absconding from Ward X and drinking and complicating her mental illness. Without proper containment of her mental health she will be at risk of disengaging from treatment.*

Alt-text: Box 3

It is important to emphasise that the picture was not uniformly bleak. As noted, 12% of the CTOs satisfied the standard we had adopted for assessing recordings of SIDMA. See Box 4 for examples.

Box 4 **Examples of formally complete responses in the recording of evidence of SIDMA.**

**Table:** 

*Ms X, by reason of her intellectual disability, is unable to comprehend any complex information including the care and treatment that is provided or the rationale for it. I am therefore of the opinion that Ms X's ability to make informed decisions about the provision of medical treatment is significantly impaired.*
*X's insight into his mental illness is variable with there being times that he does not accept he is mentally unwell. He remains very thought disordered which prevents him from understanding information about his illness and the need for treatment, and so his decision making regarding his medical treatment is significantly impaired.*

Alt-text: Box 4

## 6 Discussion

Two decades after the Millan Report, Scotland is once again in the midst of a review of the legislative framework pertaining to mental health care and treatment. While there have been many significant developments in the interim, several important constants remain. The terms of reference for the current review (hereafter: the Scott Review) reaffirm Scotland's commitment to the policy objectives of enhancing patient autonomy and non-discrimination (Secretariat to the review of the mental health legislation in Scotland, 2019). Moreover, the option of ‘fusing’ mental health and mental capacity legislation remains on the table – a cause significantly advanced by the adoption by the Northern Ireland Assembly of the Mental Capacity Act (Northern Ireland), 2016. As we have documented in Section 2, Scotland sought to navigate this policy terrain two decades ago by adopting a distinctive direction of travel, introducing SIDMA as a middle path between the status quo ante and a ‘full fusion’ approach. The current review panel will need to decide whether to continue down this path. In effect there are three broad options: to retain SIDMA unchanged, to modify SIDMA, or to abandon SIDMA.

The choice among these options must be informed by the full deliberative and consultative processes of the review and any subsequent legislative proceedings. Nonetheless, the findings that emerged from our analysis are certainly relevant to the policy decision facing the Scott Review, and have a broader relevance for other jurisdictions that are reconsidering the legal threshold for the use of psychiatric compulsion.

In considering the relevance of our findings to these policy options, we note first that we found evidence that SIDMA is indeed fulfilling some of the original purposes envisioned in the Millan Report. As we have seen, SIDMA was introduced in part because of concerns about possible unintended consequences that might result from a ‘full fusion’ approach. At the time of the Millan Review, the Royal College of Psychiatrists in Scotland expressed concern that people with eating disorders, mood disorders, or obsessive-compulsive disorder might be excluded from treatment by a capacity test (Millan, 55). Moreover, because capacity tests are time-specific, there was concern that patients exhibiting fluctuating capacity might be excluded from eligibility for compulsory treatment under a fusion approach, despite being in urgent need of care (Millan, 56). As regards these concerns, we found evidence that SIDMA is indeed working as intended, introducing consideration of decision-making ability into the CTO process while nonetheless facilitating detention of persons in all these categories.

However, our analysis of CTOs also uncovered evidence that raises serious concerns about SIDMA-assessment and SIDMA-reporting in current psychiatric practice. As we have seen, 5% of the CTOs that we analysed failed to record any evidence that SIDMA was present. Twelve percent based the finding of SIDMA entirely on an unelaborated claim that the patient lacks insight. Eighty-eight percent of the CTOs failed to meet the minimum criteria for formal completeness in the reporting of SIDMA, with assessors frequently failing to identify a link between the person's mental disorder and their impaired decision-making ability, as required by law. Moreover, we found evidence indicating that the readability of the SIDMA fields in CTOs was often poor. Conversely, we found little evidence of steps taken to provide support for those whose decision-making abilities was impaired.

How can these shortcomings best be explained and remedied? The results from our analysis do not provide sufficient evidence to answer these questions definitively, but it is worth surveying some possibilities. A natural starting point is the existing guidance for practitioners who must apply the SIDMA standard. As we have seen, there is a no shortage of SIDMA guidance in the *Training Manual*, in the *Code of Practice*, and in publications from the Commission. If nothing else, this guidance could and should be reviewed and updated to ensure greater consistency and clarity. As we have seen, the existing guidance is not entirely univocal, and some of the most recent guidance has been formulated only for use in the context of eating disorders, which comprises only a tiny portion of the relevant population. So a review and updating of guidance would certainly be a welcome step.

We are doubtful, however, that revised guidance alone will suffice to ensure high quality SIDMA-assessment. It is important to appreciate that many of the failings identified in the present study constitute clear *departures* from existing guidance. At the very least, new guidance would need to be combined with a new training curriculum on SIDMA and more robust quality-control mechanisms in the process of assessing applications for CTOs. But we must also consider the possibility that part of the problem lies in the underlying legislation itself. As we noted at the outset, one striking feature of the SIDMA provisions in the Act is that they leave the concept of SIDMA undefined. Moreover, the statute's reliance on the term ‘significantly’ introduces an unavoidable element of vagueness into the current legal standard. The statute itself therefore fails to project any particular structure for the assessment of SIDMA, leaving assessors themselves with considerable latitude in reaching a determination as to its presence or absence.^12^ One possible response to the current shortcomings in practice would be to adopt a statutory definition of SIDMA that could be used to inform new guidance and to structure training, assessments and reporting. Doing so would have the additional advantage of marking a clear distinction between the SIDMA standard and the concept of mental capacity defined in the AWIA.

Whether in reviewing guidance and training, or in contemplating statutory reform, the data from our study show the need for greater clarity on the relationship between impaired insight and SIDMA. Taken together, the Shek et al. study and our own data clearly demonstrate that impaired insight is consistently the single most commonly cited ground for a finding of SIDMA. In a significant subset of cases, insight is the *only* ground offered in support of such a finding. Because SIDMA lacks a statutory definition, there is no clear way to determine whether impaired insight alone is a legally sufficient warrant for a finding of SIDMA. Existing guidance on this question is less clear than it might be. In particular, greater clarity is needed on the difficult question of whether a patient's disbelief of diagnostic or treatment information itself suffices to establish that their ability to make a treatment decision is significantly impaired.^13^

## 7 Limitations

The present study was based on an analysis of administrative records associated with CTOs. We did not study the assessment of SIDMA in emergency or short-term detentions. We did not undertake to study the processes used by assessors in preparing such applications, nor the scrutiny undertaken of the SIDMA-reporting at the person's Mental Health Tribunal hearing. A fuller consideration of the role of SIDMA-assessments in authorising coercion would need to incorporate a thorough examination of these further processes. In examining CTOs, we extracted the SIDMA data from the larger CTO applications, leaving the possibility that relevant information contained in the CTO was not considered in our analysis. For our assessment of readability, we used a metric that was simple to apply but considers only a limited number of factors such a sentence length and syllables-per-word. We did not undertake to determine the extent to which this metric is a valid proxy for understandability by mental health patients or those who support them. Finally, the normative measure that we used in our quality assessment was confined to an assessment of the formal completeness of the SIDMA-reporting; we had no way of determining in any particular case whether SIDMA was actually present or absent in the person being assessed.

## 8 Conclusion

SIDMA is a concept unique to Scots law, representing a distinctive strategy in ongoing efforts to reform mental health legislation. The reliance on SIDMA in existing Scottish mental health legislation can be understood as a compromise position in the ongoing debates about fusion law. SIDMA incorporates an assessment of a patient's decision-making abilities into the procedure for authorising compulsion. However, it stops short of outlawing coercive treatment of patients who retain the mental capacity to make a treatment decision for themselves. To the best of our knowledge, the present study is the most comprehensive analysis of how clinicians report SIDMA. Our analysis replicates and expands on work conducted ten years ago by Shek et al. (2010). That earlier study found that SIDMA was poorly recorded on reports for CTOs; our analysis shows that this is still the case. Beyond the fact that clinicians seemed to list more reasons for SIDMA, there did not appear to be a great difference in the content of their descriptions, despite the fact that the SIDMA standard has now been in effect for more than 15 years.

In coming to a considered view about SIDMA, attention should be given both to its promise and to the pitfalls that have manifested themselves in practice. Part of the original promise of SIDMA was to provide enhanced respect for patient autonomy in decisions about psychiatric compulsion. The strategy for doing so was to incorporate an assessment of patients' ability to make care and treatment decision for themselves, reserving the recourse to compulsion for those whose ability to make such decisions was significantly impaired. But the lack of a statutory definition of SIDMA has been a pitfall on the path of implementation. In the absence of a definition that would project a structured framework for the assessment of SIDMA, the record suggests that SIDMA has not proven to be a robust tool for ensuring enhanced respect for patient autonomy. The Scottish experiment with SIDMA therefore presents valuable lessons for jurisdictions around the world which are considering reform strategies which retain provision for use of psychiatric compulsion while ensuring greater respect for patient autonomy and mitigating concerns about potential discrimination.

## Funding

This research was funded in whole, or in part, by the 10.13039/100010269Wellcome Trust [203376/Z/16/Z]. For the purpose of open access, the author has applied a CC BY public copyright licence to any Author Accepted Manuscript version arising from this submission.

## Declaration of Competing Interest

AC is a member of the subgroup on capacity and supported decision making of the Scottish Mental Health Law Review. GM is a member of the Executive Team of the Scottish Mental Health Law Review.

## Appendix A Supplementary data

Supplementary material Image 1

## Footnotes

2 No other jurisdiction of which we are aware includes the SIDMA requirement found in Scots law. For a survey of mental health legislation with similar provisions, see Cronin, Gouda, McDonald, and Hallahan (2017). Perhaps the closest variant on SIDMA is the broader ‘impaired judgement’ requirement that figures in *sec*. 3(1)(b)(i) of the *Mental Health Act (Republic of Ireland) (2001)* in the Republic of Ireland.

3 The Millan Commission first met in March 1999; the devolved Scottish Parliament opened in May. The final report of the Millan Commission was issued in January 2001.

4 For an analysis of the reasons for detention provided in medical reports under the 1984 Act in practice, see, Bean, McGuckin, and MacPherson (2000), ch 5.

5 For discussion of fusion law, see Szmukler & Holloway, 2000; Szmukler, Daw, & Dawson, 2010; Szmukler, 2017

6 The rationale for the change from ‘judgement’ to ‘decision-making ability’ was articulated in the Scottish Executive's policy statement that was issued in advance of consideration of the bill by the Scottish Parliament. See Scottish Executive, 2001b, para. 3.6.

7 According to the AWIA, “‘incapable’ means incapable of— (a) acting; or (b) making decisions; or (c) communicating decisions; or (d) understanding decisions; or (e) retaining the memory of decisions, as mentioned in any provision of this Act, by reason of mental disorder or of inability to communicate because of physical disability” (AWIA 1(6)).

8 The first sentence of this explanation echoes language from Scottish Executive (2001b para. 3.7). The claim that SIDMA ‘arises out of mental disorder alone’ should be understood in light of *sec*. 328(1) of the Act, which defines a mental disorder as any mental illness, personality disorder or learning disability, ‘however caused or manifested.’

9 See e.g. the exchange between Guidry-Grimes (2019) and David and Ariyo (2021).

10 These percentages add up to more than 100% because 16 forms analysed by Shek et al. cited more than one grounds for the finding of SIDMA. At one point in their paper, Shek et al. report that they ‘found that the most common reasons for SIDMA were lack of insight (44%)’ (Shek et al., 2010, p.241). But this statistic excludes cases where insight was cited as one among several grounds for a finding of SIDMA.

11 In England and Wales, the National Institute for Health and Care Excellence (NICE) made a similar recommendation: “If a practitioner believes a person's insight/lack of insight is relevant to their assessment of the person's capacity, they must clearly record what they mean by insight/lack of insight in this context and how they believe it affects/does not affect the person's capacity” (National Institute for Health and Care Excellence, 2018: 25).

12 Cairns et al. (2005) have demonstrated that use of a structured approach in the assessment of decision-making capacity results in higher interrater reliability.

13 Guidance from the Commission does not include ‘believing information’ among the components of the decision-making process (Mental Welfare Commission for Scotland, 2017b: 13). By contrast, both the *Training Manual* and the *Code of Practice* for the Act specifically make reference to believing information in their discussion of SIDMA. The *Training Manual* indicates that ‘SIDMA occurs when a mental disorder affects the person's ability to *believe*, understand and retain information, and to make and communicate decisions’ (Scottish Executive, 2005a, 45; emphasis added). The *Code of Practice* describes factors to be considered in the assessment of SIDMA as including the ‘ability to *believe*, understand and retain information concerning their care and treatment’ (Scottish Executive, 2005b, para 1.22; emphasis added). While these various claims are not strictly inconsistent, they do not add up to a clear account of how a patient's lack of acceptance of illness should be factored in to an assessment of SIDMA.
