# Supplementary material for: SIDMA as a criterion for psychiatric compulsion: An analysis of compulsory treatment orders in Scotland
Source: Int J Law Psychiatry. 2021 Sep-Oct;78:101736. doi: 10.1016/j.ijlp.2021.101736 (PMC8527859; doi:10.1016/j.ijlp.2021.101736)
Supplement: Supplementary file 1 — Supplementary material [file mmc1.docx]

**Supplementary File – Coding framework**

| **Section** | **Main concept** | **Question** | **Answer** |
| --- | --- | --- | --- |
| 1 | Lack of insight | 1. Does the application note the patient to have a lack of insight either by mentioning ‘insight’ or by describing a lack of it? | Y/N |
|  |  | 2. Does the application use the keyword ‘insight’? | Y/N/NA |
|  |  | 3. If keyword ‘insight’ is used, does the application give an explanation for what lack of insight means? | Y/N/NA |
|  | Confusion/cognitive impairment | 4. Does the application describe if the patient is confused or has any evidence of cognitive impairment? | Y/N |
|  | Psychotic symptoms | 5. Does the application state that the patient has psychotic symptoms? | Y/N |
|  | Other | 6. Does the report give any other reason for SIDMA?  7. Note reason given for Q6 (if yes) | Y/N  Free text |
| 2 | Insight | 8. Does the application describe the patient as lacking insight either by mentioning ‘insight’ or by describing a lack of insight?  9. If yes to 8, does the application describe how this lack of insight affects the patient’s decision making ability? | Y/N  Y/N/NA |
|  | Non-compliance | 10. Does the application describe the patient as being non-compliant with their treatment? | Y/N |
|  |  | 11. Does the application use one of the following in their description of/reference to non-compliance? Either (non)-compliance or (non)-concordance | Y/N/NA |
|  |  | 12. Does the application give a reason for the non-compliance which is a consequence of mental illness? | Y/N/NA |
|  | Confusion/cognitive impairment | 13. Does the application describe if the patient is confused or has any evidence of cognitive impairment?  14. If yes to 13, does the application describe how this confusion or cognitive impairment affects the patient’s decision making ability? | Y/N  Y/N/NA |
|  | Mental illness | 15. Does the application describe symptoms of mental illness?  16. If yes to 15, does the application describe how these symptoms affect the patient’s decision making ability? | Y/N  Y/N/NA |
|  | Learning disability | 17. Does the application describe symptoms of a learning disability?  18. If yes to 17, does the application describe how these symptoms affect the patient’s decision making ability? | Y/N  Y/N/NA |
|  | Personality disorder | 19. Does the application describe symptoms of a personality disorder?  20. If yes to 19, does the application describe how these symptoms affect the patient’s decision making ability? | Y/N  Y/N/NA |
|  | Other | 21. Does the application give any other reason which interferes with the person’s decision making ability? (If yes describe this reason)  22. If yes to 21, does the application describe how this other reason affects the patient’s decision making ability? | Y/N  Y/N/NA |
|  | Decision making process/capacity | 23. Does the application state that the patient is not able to perform elements of the decision making process (act, make, communicate, understand, retain).  24. If yes to 23, does the application give a reason the patient is unable to do this? | Y/N  Y/N/NA |
|  | Inconsistency | 25. Does the application describe the patient as exhibiting inconsistency, for example between what the individual says and the way she/he behaves? | Y/N |
| 3 | Symptoms | 26. Does the application describe which features of the condition are interfering with decision-making? | Y/N |
|  | Decision-making process | 27. Does the application make specific reference to which parts of the decision-making process (act or make or communicate or understand or retain) are impaired? | Y/N |
|  | Link | 28. If yes to 26 and 27, does the application make a link between the features of the condition which interfere with decision-making and the parts of the decision-making process with which they interfere?  29. If there is a link between the features of the condition which interfere with decision-making and the parts of the decision-making process with which they interfere, does the clinician give an overall summing up/conclusion that the patient’s decision making is impaired? | Y/N/NA  Y/N/NA |
|  | Inconsistency | 30. Does the application describe the patient as exhibiting inconsistency, for example between what the individual says and the way she/he behaves? | Y/N |
